# Elaborating on Immune Cells in Aging and Alzheimer's Disease with Single‐Cell Long‐Read Sequencing

**DOI:** 10.1002/alz70855_100419

**Published:** 2025-12-23

**Authors:** Benney MR Argue, David Gate

**Affiliations:** ^1^ Northwestern University, Chicago, IL, USA; ^2^ Northwestern University Feinberg School of Medicine, Chicago, IL, USA

## Abstract

**Background:**

Single‐cell long‐read sequencing was performed on immune cells from cerebrospinal fluid (CSF) and blood to assess isoform diversity in healthy aging individuals and those diagnosed with mild cognitive impairment (MCI) or Alzheimer's disease (AD).

**Method:**

cDNA from single‐cell experiments was subjected to long‐read sequencing using Oxford Nanopore Technologies. The dataset included immune cells from CSF (45 controls, 13 MCI/AD) and blood (22 controls, 28 AD). Computational analysis incorporated scNanoGPS for extracting cell barcodes and mapping reads to the genome, IsoQuant for isoform modeling, and SQANTI3 for filtering artifacts.

**Results:**

Single‐cell long‐read sequencing identified 97,920 unique transcripts, with half of all genes exhibiting multiple isoforms. Notably, 20% of the detected isoforms were previously unannotated in the human genome. Novel isoforms were observed in AD‐associated genes, including *BIN1* and *PTK2B*, with *BIN1* displaying lymphoid cell‐specific isoforms.

**Conclusion:**

Human immune cells demonstrate extensive isoform diversity, including previously unannotated isoforms, particularly in genes implicated in AD.

